# Evaluating the feasibility of a pharmacist-guided patient-driven intervention to improve blood pressure control in patients with CKD

**DOI:** 10.1186/s40814-019-0410-0

**Published:** 2019-02-12

**Authors:** Charles Hopley, Emily Andrews, Patrick Klem, Michelle Jonjak, Ann Grothe, Patrick Ten Eyck, Zhiying You, Sarah J. Billups, Corey Lyon, Korey Kennelty, Bradley Dixon, Diana Jalal

**Affiliations:** 1 Division of Renal Diseases and Hypertension, University of Colorado Anschutz Medical Center, Aurora, CO USA; 2 Kidney and Hypertension Clinic, University of Colorado Hospitals, Aurora, CO USA; 3 Institute for Clinical and Translational Science, University of Iowa, Iowa City, IA USA; 4 University of Colorado Skaggs School of Pharmacy and Pharmaceutical Sciences, Anschutz Medical Center, Aurora, CO USA; 5 AF Williams Family Medicine Center at Stapleton, University of Colorado Anschutz Medical Center, Aurora, CO USA; 6 Department of Pharmacy Practice and Science, College of Pharmacy, University of Iowa, Iowa City, IA USA; 7 Department of Family Medicine, Carver College of Medicine, University of Iowa, Iowa City, IA USA; 8 Nephrology Division, Carver College of Medicine, University of Iowa, 200 Hawkins Dr., E300C-GH, Iowa City, IA 52242 USA

**Keywords:** Blood pressure, Adherence, Implementation science

## Abstract

**Background:**

Self-titration of blood pressure (BP) medications and lifestyle modifications are effective and safe strategies to lower BP. We assessed the feasibility of implementing a pharmacist-guided, patient-driven self-titration protocol and standardized dietary counseling to improve BP in the chronic kidney disease (CKD) clinic.

**Methods:**

Adult patients seen in the CKD clinic were identified via registry screening. Inclusion criteria were as follows: a diagnosis of hypertension, average of the last 3 office BP > 150/90 mmHg, and prescribed 3 or fewer BP medications. Patients with severe hypertension were excluded. BP goals were established and patients were referred to the clinical pharmacist who provided them a BP cuff, a BP medication titration plan (based on home BP monitoring), and dietary education. The following outcomes were evaluated: appeal of the program to patients identified by the registry, patient adherence to the protocol and 6-month office BP, and provider attitudes and acceptance of the protocol.

**Results:**

Seventeen patients enrolled in the pilot, the majority recruited via clinic schedule screening. Eleven of the 17 patients completed a 6-month office follow-up visit. Three of the 11 patients met their pre-specified office BP goal. There was, however, significant improvement in 6-month office systolic and diastolic BP. Twelve of 17 patients were adherent to entering home BP in EMR. Provider satisfaction with the protocol was high.

**Conclusion:**

Our preliminary data suggest that patient-driven self-titration of BP medications is feasible and well received by providers. Future studies are needed to validate these findings and to evaluate the safety and efficacy of this approach.

## Introduction

Hypertension is one of the most important risk factors for renal and cardiovascular morbidity and mortality [1]. Despite advances in therapy strategies and attention placed on target blood pressure (BP) goals, only about two thirds of treated patients reach their pre-specified BP goal [2]. New trials demonstrate the benefits of stricter BP control, and the most recent guidelines (released by the American Heart Association) reflect the overwhelming benefit of tighter BP control demonstrated in these trials [3, 4]. As goal blood pressures are lowered, it will be harder for patients to attain the target BP [3].

The population cared for in the chronic kidney disease (CKD) clinic presents a special challenge in terms of hypertension management. First, patients CKD often have hypertension secondary to their underlying kidney disease and are more likely to be resistant to first-line therapy than individuals without CKD [5]. Second, patients with CKD are known to be at elevated cardiovascular risk compared to the general population, making BP control all the more important for this group of patients and BP goals more stringent, particularly in those with glomerular disease [6, 7].

In most practices, licensed providers perform the bulk of hypertension management. This is despite evidence of the effectiveness of patient-dependent self-monitoring and titration protocols [8–12]. Such approaches engage patients in their care and eliminate the lag time inherent in waiting for the next appointment to intensify therapy in response to uncontrolled BP [8, 9]. Taking this a step further, self-management of hypertension consisting of self-monitoring of home BP and the implementation of a simple pre-determined titration plan by the patient has been shown to be more effective at lowering systolic BP than the usual care [11, 12]. Additionally, well-conducted studies have demonstrated the importance of dietary approaches to augment BP control [13, 14]. While the Dietary Approach to Systolic Hypertension (DASH) diet has become popular in niche groups for dieters, published data in the last decade indicate low rates of lifestyle modification counseling by providers for patients with hypertension worldwide, being less than 50% in the majority of reports [15–19].

The results of clinical trials have found limited implementation into clinical practice, and it remains unclear whether such interventions are feasible in clinical practice. Furthermore, the recent governmental push to increase use of electronic medical record (EMR) systems which have the capabilities to pool data and create specific population registries present an opportunity to modify the health of a patient population at large and to evaluate whether identifying patients via EMR screening is feasible and/or effective. The overall objective of this pilot project was to evaluate the feasibility of implementing an interdisciplinary approach for the management of BP in a CKD clinic. Specifically, we evaluated the feasibility of implementing a pharmacist-guided patient-driven approach for self-monitoring and self-titration of BP medications and the integration of dietary counseling as a routine component of BP management in a CKD clinic.

## Materials and methods

### Subjects and setting

The pilot took place in the CKD clinic, a subspecialty multidisciplinary clinic of 25 providers including physicians and advanced practice providers, at the University of Colorado Anschutz Medical Center. The proposal was funded by a Clinical Effectiveness and Patient Safety Small grants program, an institutional grant awarded after a peer review process. An application was submitted for approval of the project to the Institutional Review Board. Considering the nature of the intervention and the setting, this was approved to proceed as a quality improvement initiative. Since this was approved as a quality improvement initiative, informed consent was not required or obtained.

The CKD clinic at UCH is staffed by a clinical renal nurse, a dietician, and a clinical pharmacist. In 2015, 718 patients were identified as hypertensive in the CKD clinic. Of these, the most recent blood pressure was < 140/90 mmHg in only 423 (59%). This cutoff was chosen based on the JNC 8 guidelines, which were the most recent guidelines for goal BP at the time of the protocol design [20]. The CKD clinic BP management follows a traditional approach, medication adjustments are made by the provider during the clinic visit, and future titrations hinge on future face-to-face follow-up visits with the provider. The CKD clinic utilizes Epic EMR, and the patients are offered access to the patient portal *(My Health Connection*, EPIC propriety).

Adult patients seen in the CKD clinic between August 1, 2015, and August 1, 2016, were eligible to participate if they had a diagnosis of hypertension and an average of the last 3 office BP readings > 150/90 mmHg. Additionally, the inclusion criteria required the following: potential participants were receiving three or fewer BP medications (in order to allow for up-titrations) and the participants were willing to participate. Exclusion criteria included the following: BP > 200/100 mmHg, life-limiting illness, or dementia.

### Intervention

Our protocol was adapted based on studies by McManus et al. that showed patient self-titration of BP medications was effective and safe in patients with high BP including those with high-risk conditions such as underlying cardiovascular disease or CKD [11, 12]. Initially, patients were identified for participation by screening EPIC CKD or HTN registry reports in *HealthyPlanet*. The following criteria were applied in the search: (1) patients seen between 8/5/15 and 8/5/16 in the department “AMC KID HTN”, (2) patient status not equal to deceased, (3) age 18–85 years, and (4) last DBP ≥ 90 mmHg and last SBP 150–199 mmHg. When patients were contacted, their interest in participation and ability to interact with the patient portal *My Health Connection* was assessed. The primary renal provider (physician or advanced practice provider) was alerted of participation and cosigned the order to initiate the patient’s first visit with the clinical pharmacist.

Patients who agreed to participate were scheduled for a 60-min visit with the clinical pharmacist with the following objectives: devise an individualized titration plan, educate the patient on correct BP measurement technique and how to record readings in *My Health Connection*, and provide an introduction to DASH diet. Additionally, all patients with estimated glomerular filtration rate (eGFR) < 60 ml/min/1.73m^2^ were offered a referral to the registered dietician within the CKD clinic. Patients were provided their own automated BP cuffs. We used the A&D Medical model # UA-611 BP monitor (https://medical.andonline.com/product/blood-pressure-monitor/ua-611).

The clinical pharmacist utilized the Collaborative Drug Therapy Management protocol [21] to develop and implement an individualized therapeutic plan for each patient. They developed an algorithm for each patient aimed to maximize the dosage of each medication in a stepwise fashion and then to add an additional agent in a rational order to exhaust first-line therapy prior to advancing to second- and third-line agents. Patients were assigned a home BP goal, which was based on personal risk factors and best evidence available [4, 22, 23]. Patients were instructed to measure their BP a minimum of 6 times per week [10] and record it into *My Health Connection*, which was accessible to both the patient and the clinical pharmacist. A medication titration was triggered if greater than 30% of measurements were above goal in a 2-week interval [24]. We anticipated home BP should be 5 mmHg lower than office BP. Hence, if goal office BP is < 140/90 mmHg, then medication adjustments would be prompted by 3 out of 10 home BP readings > 135/85 mmHg [25]. Patients would communicate with the pharmacist via EMR when a titration had been made and the pharmacist would update the medication list and order the appropriate follow-up labs if needed. When patients were unable to communicate via EMR, they would communicate with the clinic nursing staff via telephone or email and the clinic nurse would enter a note in EMR to notify the pharmacist.

The dietary information given to patients was scripted. If a patient’s eGFR was greater than 60 ml/min/1.73m^2^, the information was based on DASH principles. As DASH places a high emphasis on high potassium foods, patients with eGFR < 60 ml/min/1.73m^2^ were offered a visit with a dietitian and provided information on low sodium strategies without the emphasis on high potassium.

### Outcomes and measures

The following outcomes were evaluated: appeal of the program to patients identified by the *HealthyPlanet registry*, patient adherence to the protocol, and provider attitudes and acceptance. We intended to evaluate the appeal of the program to patients as proportion of patients (identified by the registry*)* who agreed to participate. Patient adherence was evaluated as the number of participants that entered their home BP readings by *My Health Connection* and number of participants that presented for the 6-month follow-up visit. In addition, we evaluated the number of BP entries per patient. Office BP was evaluated at baseline and at the 6-month office visit. CKD provider appeal was evaluated as the number and % of providers who accepted the pended order for referral to the clinical pharmacist. Additionally, CKD provider attitudes toward this approach were assessed utilizing a 17-item survey both before the project started and after 9 months of project run time. The surveys were anonymous and conducted using the Survey Monkey platform as this allowed the team to create an online survey with basic computer skills without additional cost [26]. Secondary outcomes included number of medication titrations or initiations performed. Additionally, we evaluated the following secondary exploratory outcomes: office BP at 6 months, BP change from baseline, and the number (%) of patients who achieved pre-specified BP goals.

The following process matrices were evaluated: the % of participants seen by the clinical pharmacist and given a detailed titration plan, the % of participants given dietary education or offered a formal dietary consult, and the % of participants provided with a BP cuff and given instructions to manually enter readings into the interactive function of the EMR.

### Patient safety monitoring

Patients were contacted 3 months into participation and asked about the occurrence of any adverse events that could be associated with their antihypertensive therapy. This phone call was conducted by the renal nurse and documented in EMR and sent to the clinical pharmacist. Additionally, after 6 months of participation, a follow-up clinical visit was scheduled with the clinical pharmacist to assess progress.

### Data analysis

For descriptive statistics, mean ± SD are provided for continuous variables (e.g., BP) and proportion for categorical variables. The following variables were evaluated: proportion of patients achieving BP goal, proportion of patients who agreed to participate, number of participants that entered their home BP readings by *My Health Connection* at the designated intervals, and number of participants that presented for the 6-month follow-up visit. In addition, the number and percentage of providers who accepted the pended order for referral to the clinical pharmacist was assessed. The paired *t* test was used in pre-post comparison of BP, and the McNemar exact test was performed as appropriate in comparison of dichotomized variables with pre-post BP measures, without pre-specified statistical power because of the pilot and exploratory nature of the study. Additionally, we utilized non-parametric testing (Spearman’s rank) to explore the potential correlation between the number of BP entries in EMR and the change in office BP at the 6-month follow-up visit. All statistical analyses were conducted using SAS version 9.4 (SAS Institute, Cary, NC).

## Results

### Appeal of the program to patients identified by registry screening

**Fig. 1:**
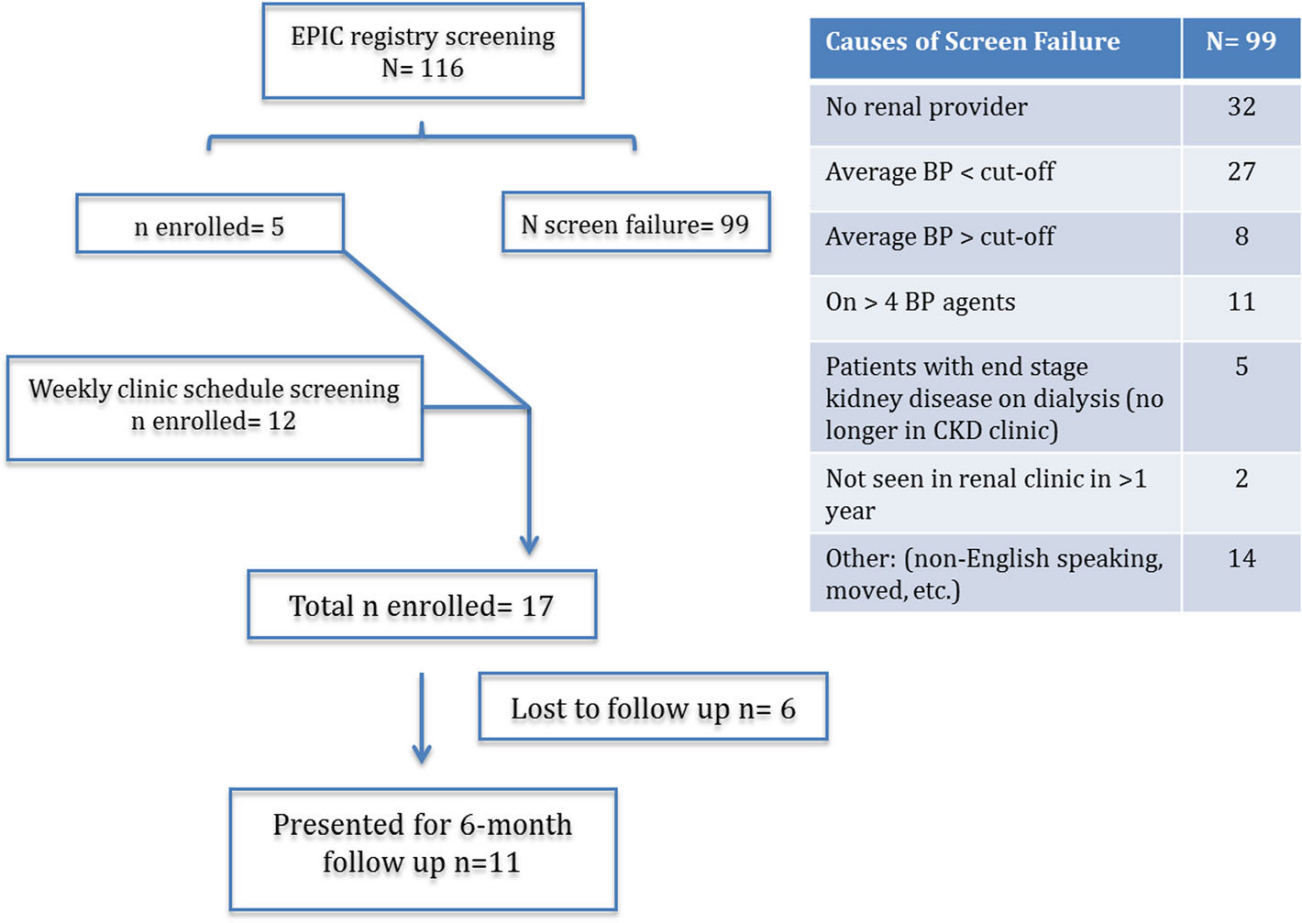
Flow chart for recruitment

Utilizing the initial search strategy, we screened the CKD and HTN registries within the EMR for potential participants. Based on the criteria outlined for the search, a total of 116 patients were identified as potential participants. Of those, 99 patients were excluded. The reasons for exclusion are shown in Fig. 1. Of note, 32 patients were excluded due to no provider in our clinic and 11 patients due to being on > 3 BP medications. Of the 19 potential participants, 5 (26%) were interested and subsequently participated in the program. Despite extensive effort to refine search filters (including seeking counseling of the EMR specialists within our institution), no further participants were recruited using this strategy. The 12 patients who subsequently agreed to participate were recruited via the clinic schedule screening process. Briefly, potential participants were identified by screening future clinic visits. Upcoming provider clinic schedules were audited by the renal nurse, and eligible patients were identified based on the same criteria above. Participants were approached by the clinical renal nurse during the clinic visit, and if interested, an order for the protocol was placed by the renal nurse. Utilizing this strategy, 12 patients agreed to participate. Baseline characteristics for all the participants are presented in Table 1.

**Table 1:** Baseline characteristics for all participants

Age, mean years (SD)	54.4 (19.85)
Female sex, *n* (%)	9 (60)
Black race, *n* (%)	8 (53)
Systolic blood pressure, mean mmHg (SD)	154 (16)
Diastolic blood pressure, mean mmHg (SD)	88 (11)
Diabetic, *n* (%)	7 (46)
eGFR, mean ml/min/1.73 m^2^ (SD)	55 (25)
eGFR < 60 ml/min/1.73 m^2^, *n* (%)	9 (60)
Number of antihypertensive agents (mean)	2.4
Antihypertensive class, *n* (%)	
ACEi/ARB	11 (65%)
Calcium channel blocker	10 (59%)
Diuretic	9 (53%)
β-blocker	4 (24%)

### Patient adherence and office BP at 6 months

Twelve of the 17 patients (70%) were adherent in entering their BP readings into EMR. In addition, 11 of the 17 patients had follow-up visits with the clinical pharmacist at 6 months. Only 3 of the 17 patients (18%) met their pre-specified office blood pressure goal at the 6-month follow-up visit (the McNemar exact test *p* value was 0.32 and 0.18 for systolic and diastolic BP, respectively). Although most of the patients who attended the 6-month follow-up visit did not achieve the pre-specified office BP goal, there was significant improvement in office systolic and diastolic BP (paired *t* test *p* value was 0.02 and 0.01 for systolic and diastolic BP, respectively). Data for the 11 participants who had 6-month visits are shown in Table 2. Non-parametric analysis indicated that the number of BP entries in EMR tended to correlate with greater decline in 6-month office systolic BP (correlation coefficient − 0.44, *p* value = 0.18). We found no such correlation between the number of BP entries and diastolic BP. Six of the 12 patients who were adherent with entering their blood pressure values achieved pre-specified BP goals at home. Similarly, 3 patients had improvement in their home blood pressure averages, despite not achieving goal (data for home BP not shown). Of note, a total of eight medication up-titrations were made. Additionally, 15 new medications were added to regimens as prescribed by the titration plan. There were two down-titrations made, and 3 medications were discontinued.

**Table 2:** Patient study data

Study ID #	Sex	Age (years)	Race	Baseline BP	6-month BP	Change in SBP*	Change in DBP*	BP entries
1	M	56	Caucasian	168/112	140/82	− 28	− 30	72
11	F	72	Caucasian	123/66	145/66	22	0	7
12	M	75	Caucasian	164/78	138/68	− 26	− 10	37
13	M	19	AA	148/108	122/71	− 26	− 37	1
17	M	34	AA	151/88	139/97	− 12	9	54
19	F	93	AA	174/83	148/94	− 26	11	66
20	F	73	Caucasian	184/80	138/62	− 46	− 18	49
21	F	37	Caucasian	136/89	133/88	− 3	− 1	56
22	F	76	AA	165/84	144/70	− 21	− 14	27
23	M	53	Caucasian	152/90	152/74	0	− 16	23
24	F	34	AA	156/84	161/79	5	− 5	32

*M* male, *F* female, *AA* African American

*P value < 0.05. Specifically, paired *t* test *p* value was 0.02 and 0.01 for systolic and diastolic BP, respectively

### Provider attitudes and acceptance of the protocol

All but one of the placed referrals for pharmacist management of hypertension were signed by the CKD provider. The order that was not signed was the first one placed and did not appear as a pended order for the provider requiring a change to the order placement procedure. Pre- and post-implementation surveys of providers within the renal clinic demonstrated that initially providers favored the idea of pharmacy-guided, patient-driven titration approaches. Sixteen providers (out of 25) replied to the pre-implementation survey and 12 to the post-implementation survey. The post-implementation survey results demonstrated that provider support increased further during the program. Please see selected results from surveys in Table 3.

**Table 3:** Provider surveys

		Strongly agree	Agree	Disagree	Strongly disagree
The pharmacy service supports clinical decision-making for management of BP medications	Post	9	3	0	0
	Pre	6	9	0	1
Pharmacist-guided patient-driven up-titration of BP medications improves physician and provider satisfaction	Post	8	4	0	0
	Pre	4	11	0	1
Lifestyle and dietary modifications are an integral part of BP management and should be applied routinely in clinical practice	Post	10	2	0	0
	Pre	13	3	0	0

### Process measures

All of the enrolled patients (100%) were given a detailed titration plan. All of the enrolled patients (100%) were given dietary education, and 100% were offered referral for formal dietary consult. Similarly, 100% of patients were provided with a BP cuff and given instructions to manually enter readings into the interactive function of the EMR. An issue we encountered initially was patients’ inability/lack of experience with using the EMR patient portal *My Health Connection*, despite providing the instructions. We subsequently planned on the clinical pharmacist educating patients on entering their BP in EMR. However, this was not possible as some patients did not have access to their EMR patient portal function. We subsequently provided a letter of instructions to the patients regarding the importance of establishing an access account for the portal prior to visit with the clinical pharmacist. Once that was implemented, we noted a significant improvement in adherence as reported above.

### Safety

One patient had symptomatic hypotension subsequent to the prescribed medication up-titration plan. This patient communicated with the CKD clinic nursing staff, and their medications were withheld leading to resolution of symptoms without further intervention. The clinic nursing staff entered a note in epic and notified the clinical pharmacist per the protocol. We observed no other adverse events.

## Discussion

We have demonstrated, for the first time, that pharmacist-guided patient-driven titration of BP medications is feasible and well-received by providers in a subspecialty clinic at a major academic medical center. Importantly, our data suggest that such a program improves BP control. While a small proportion of patients met their pre-specified office BP goal at the 6-month visit, a much greater proportion of patients demonstrated improvement in their BP readings. One lesson from recent studies is that the general improvement in BP offers significant benefits even when patients do not achieve their pre-specified goal [4]. As such, our findings, while preliminary, provide a potentially novel approach that could have a significant impact on the management of high BP in clinical practice.

Our protocol was adapted based on studies by McManus et al. that showed patient self-titration of BP medications was effective and safe in patients with high BP including those with high-risk conditions such as our patient population [11, 12]. However, no studies to date have evaluated the feasibility of implementing this approach in a clinical practice setting. In addition, in the studies by McManus et al., the physician outlined the BP medication plan for the patients, whereas in our pilot, we utilized the clinical pharmacist. We elected this approach based on data that pharmacist/physician co-management of blood pressure is associated with improved BP lowering [27, 28]. As such, although our protocol was adapted based on previous studies, it is novel in and of itself due to the utilization of a broader group of interdisciplinary team members including the provider, the clinical pharmacists, the clinic dietician, and the patient.

Our protocol was not designed to measure adherence with dietary counseling or with medications. As such, one might argue that the improved BP readings in our patients represent improved adherence with either or both. However, all but one patient experienced intensification of their medication regimen per their titration plan. Though speculative, our approach likely led to BP control more rapidly than the traditional practice of titrating BP medications in clinic during face-to-face visits. Evaluating the time needed to achieving BP target was beyond the scope of this pilot. However, based on our findings, we believe that future studies designed to evaluate the effectiveness of this approach should evaluate this outcome.

Implementing our pilot as a quality improvement project allowed us to adapt our approach to better understand what would be feasible in our clinical setting. For example, our initial plans to utilize search functionality of pre-existing CKD and hypertension registries to identify potential patients did not yield as many participants as we had intended. There were several reasons for this. First, the initial screening process identified multiple patients who did not have a designated Renal Provider in our practice (a provider signature was required on the protocol order). This was despite the discrete search criteria including our clinical area. Second, it was not always readily apparent from the documentation in the patient’s medical record whether patients met enrollment criteria based on BP, and a considerable number of patients reviewed did not meet the inclusion criteria due to having either above cutoff or below cutoff BP. Third, many patients were on > 3 medications, and considering the nature of high BP in patients with CKD and the complexity of the medical regimen in this group of patients, future work should evaluate this approach in patients on more than 3 medications as well. Fourth, “cold-calling” patients identified via registry searches yielded low enrollment, possibly because some patients perceived they were being recruited to participate in a research study as opposed to utilizing a different approach for their everyday care. Patient recruitment improved with a clinic schedule-based approach that incorporated our nursing staff in a proactive screening process. This is an important finding that should be considered in future study design.

Another barrier that we encountered that limited implementation of the original plan was patients’ inability/lack of experience using the EMR patient portal as a tool to track their progress. We subsequently provided a letter of instructions to the patients regarding the importance of establishing an access account prior to the visit with the clinical pharmacist and the clinical pharmacist educated the patient the use of the interactive EMR function. This led to substantial improvement in adherence. Our findings here signal the importance of identifying other approaches for patient interface with EMR in the future. For example, the authors are aware of BP cuffs which automatically transmit data to the EMR; however, the cost was prohibitive for this pilot proposal. We ultimately achieved a high rate of adherence with self-entry of BP readings into the EMR which we believe was due to the high level of motivation by the participating patients and the dedication of our team in overcoming this barrier. In fact the high level of patient motivation may represent selection bias in the interpretation of our data. It should also be noted that self-management strategies are not ideal for every patient. Successful implementation of such strategies requires a motivated patient with some degree of health literacy. Future studies should focus on patient factors that may influence the feasibility and sustainability of such an approach. Alternatively, a more proactive approach by the clinical pharmacist may be necessary such as routine follow-up calls to evaluate BP response and adherence with the medication titration plan [29].

In addition to a motivated patient, providers must be willing to rely on the patient to assume a greater degree of autonomy with regard to medication management and on the clinical pharmacist for the monitoring of labs such as creatinine and potassium. Despite the high provider satisfaction rates with the protocol in our setting, we recognize this may not be generalizable. Some patients require the face-to-face attention gained in the traditional approach for the management of hypertension. Additionally, it is conceivable that such an approach for the management of high BP in patients with CKD may result in fewer provider visits. Due to the pilot nature of the project and the limited follow-up time, we were unable to evaluate clinic attendance times and how this may further influence the clinical practice. Further work is needed to better understand the clinical settings in which such an approach can be implemented. It is important to exercise caution in the interpretation of the exploratory efficacy outcomes reported here given the nature of the study design (to evaluate the feasibility of the intervention), the small sample size, and the paucity of data.

## Conclusions

Our findings demonstrate that implementation of a pharmacist-guided self-titration program is feasible and can lead to improved BP control in select patients. In our study, registry screening was not an effective tool for identifying patients with uncontrolled hypertension who might benefit from the developed intervention. We believe that our findings are important to inform future studies on the topic. Further work is needed to validate our preliminary data and to evaluate the safety and the efficacy of pharmacist-guided patient-driven self-titration on BP control in clinical practice.
